# TogoVar: A comprehensive Japanese genetic variation database

**DOI:** 10.1038/s41439-022-00222-9

**Published:** 2022-12-12

**Authors:** Nobutaka Mitsuhashi, Licht Toyo-oka, Toshiaki Katayama, Minae Kawashima, Shuichi Kawashima, Kazunori Miyazaki, Toshihisa Takagi

**Affiliations:** 1 Database Center for Life Science, Joint Support-Center for Data Science Research, Research Organization of Information and Systems, University of Tokyo Kashiwanoha-campus Station Satellite 6F, 178-4-4, Wakashiba, Kashiwa, Chiba 277-0871 Japan; 2 Toyama University of International Studies, 65-1, Higashi-Kuromaki, Toyama, Toyama 930-1292 Japan; 3 Department of NBDC Program, Japan Science and Technology Agency, Science Plaza, 5-3, Yonbancho, Chiyoda-ku, Tokyo 102-8666 Japan; 4 Toshiba Corporation, 1-1, Shibaura 1-Chome, Minato-ku, Tokyo 105-8001 Japan

**Keywords:** Genetic databases, Genetic variation

## Abstract

TogoVar (https://togovar.org) is a database that integrates allele frequencies derived from Japanese populations and provides annotations for variant interpretation. First, a scheme to reanalyze individual-level genome sequence data deposited in the Japanese Genotype-phenotype Archive (JGA), a controlled-access database, was established to make allele frequencies publicly available. As more Japanese individual-level genome sequence data are deposited in JGA, the sample size employed in TogoVar is expected to increase, contributing to genetic study as reference data for Japanese populations. Second, public datasets of Japanese and non-Japanese populations were integrated into TogoVar to easily compare allele frequencies in Japanese and other populations. Each variant detected in Japanese populations was assigned a TogoVar ID as a permanent identifier. Third, these variants were annotated with molecular consequence, pathogenicity, and literature information for interpreting and prioritizing variants. Here, we introduce the newly developed TogoVar database that compares allele frequencies among Japanese and non-Japanese populations and describes the integrated annotations.

## Genomic database: Genetic variation patterns in Japan

A comprehensive database of genome sequence differences found in Japanese individuals is helping researchers uncover the genetic basis of diseases occurring in the Japanese population. Nobutaka Mitsuhashi from the Database Center for Life Science in Chiba, Japan, and colleagues describe the development of TogoVar, a web-based resource that includes genetic data from more than 200,000 Japanese individuals, plus many others of non-Japanese ancestry for comparison. From the millions of tracked DNA differences, many associated with disease, researchers can search for genetic variants of interest and find information on variant frequency, clinical importance, genomic context, related publications, and more. First established in 2018, TogoVar (https://togovar.org) now provides a one-stop shop for researchers looking to interpret genomic variation data in Japanese populations.

## Introduction

The low cost of genome sequencing and genotyping has enabled the analysis of large amounts of individual-level genome sequence and genotype data, leading to the identification of disease-causing genes. Genomic data are useful and reusable for meta-analysis or confirming the results published in scientific journals. The database of Genotypes and Phenotypes (dbGaP)^1^ and the European Genome-phenome Archive (EGA)^2^ were launched as controlled-access databases in 2006 and 2008, respectively, to share individual-level data.

To promote genomic data sharing in Japanese populations, the National Bioscience Database Center (NBDC) (https://biosciencedbc.jp/en/) of the Japan Science and Technology Agency (JST) (https://www.jst.go.jp/EN/) launched the NBDC Human Database in collaboration with the DNA Data Bank of Japan (DDBJ) (https://www.ddbj.nig.ac.jp/) as a framework for sharing various data collected from human specimens in 2013. The database complies with the Personal Information Protection Law and the Ethical Guidelines for Medical and Biological Research Involving Human Subjects of Japan. The Japanese Genotype-phenotype Archive (JGA)^3^ is a controlled-access repository for the NBDC human database. Individual-level data obtained mainly from publicly funded research have been deposited in the JGA. DDBJ operates JGA by securely accepting, archiving, and delivering controlled-access data. NBDC works as a data access committee (DAC) that reviews applications for the submission and use of data. The number of submissions and uses is increasing. As of August 2022, the numbers of applications for data submission, published submissions, and applications for controlled-access data use were 411, 232, and 242, respectively. This framework realized the continuous accumulation and sharing of large individual-level genome sequence data of Japanese individuals with various genetic backgrounds.

To contribute genetic studies using deposited JGA data while protecting the privacy of the research participants, we generated original allele frequency data by reanalyzing individual-level genome sequence data deposited in JGA with the same variant calling pipeline^4^. Because the allele frequency data are no longer a code for personal identification, privacy was protected. Furthermore, as ancestry-matched controls in studies of Japanese populations, it is important to interpret the association between variants and traits precisely.

The Japanese Multi Omics Reference Panel (jMorp)^5^ and the Human Genetic Variation Database (HGVD)^6^ are well-known databases of Japanese ancestry-matched control data of allele frequency. Furthermore, the Genome Aggregation Database (gnomAD)^7^ and the Allele Frequency Aggregator (ALFA)^8^ database are frequently used as reference data because of their large sample size and diverse populations.

When using publicly available databases and our allele frequency data to interpret variation data, researchers have expressed the difficulty of collecting and integrating the allele frequency data, clinical significance from ClinVar^9^, genome-wide associations from GWAS Catalog^10^, and publications reporting variant-trait associations from PubMed. Thus, we constructed TogoVar to provide a one-stop service to obtain this information.

Here, we introduce TogoVar (aiming for data sharing by promoting the JGA database) and report on comparing allele frequencies among Japanese and non-Japanese populations.

## Materials and methods

### Variant data collection

We obtained five Japanese variant datasets, as subsequently described. Each research project obtained consent from the research participants for all the datasets. To generate the original datasets JGA-NGS and JGA-SNP, we obtained permission to process the data to improve the convenience of the use of data from submitters and aggregated individual-level genome sequence data. More information is available on the dataset page of TogoVar^11^.

#### JGA-NGS

JGA-NGS is an allele frequency dataset that aggregates individual-level genome sequence data publicly available from the JGA. Both healthy and disease groups were included. Seven WES datasets from 125 individuals were aggregated^12^.

#### JGA-SNP

JGA-SNP is an allele frequency dataset that aggregates individual-level SNP array data publicly available from the JGA. Both healthy and disease groups were included. At the time of writing, three datasets with 183,884 individuals were aggregated^13^. Most of the data were from the Tailor-made Medical Treatment Program by BBJ^14^, a biobank that enrolled research participants with 47 target diseases for 5 years starting in 2003. Clinical information was collected annually through interviews and access to medical records until 2013. DNA samples collected from research participants at baseline (2003–2008) were analyzed.

#### GEM-J WGA

GEM-J WGA is an unrestricted-access Japanese allele frequency dataset released by the Genome Medical Alliance Japan (GEM Japan) project^15^, which performed whole-genome sequencing (WGS) and joint variant calling of 7609 Japanese individuals archived in the JGA^16^. The 7609 individuals consisted of 4495 individuals from the Tohoku Medical Megabank (TMM)^17^, 2089 from the Biobank Japan (BBJ)^14^, and 257 from the RIKEN. For QC and variant calling, see hum0103-v3^18^ in the NBDC Human Database.

#### HGVD

The Human Genetic Variation Database (HGVD)^6^ is an allele frequency dataset created by whole-exome sequencing (WES) of 1208 healthy members of a community-based cohort conducted in Nagahama City, Shiga Prefecture, Japan.

#### ToMMo 8.3KJPN

ToMMo 8.3KJPN is an allele frequency dataset of 8380 individuals excluding relatives from approximately 9000 healthy volunteers sequenced in a cohort study of local residents conducted by the Tohoku Medical Megabank Project/Organization (ToMMo)^17^. In the community cohort study, 80,000 people aged 20 years and older, mainly from Miyagi and Iwate prefectures, were recruited from 2013 to 2016 and followed for at least 5 years. Individuals living in western Japan were also included in the dataset. The dataset was downloaded from the Japanese Multi Omics Reference Panel (jMorp) (https://jmorp.megabank.tohoku.ac.jp/)^5^. The variant calling method was the same as 3.5KJPN^19^, which was released before 8.3KJPN.

### Variant data processing

#### Variant calling of the JGA-NGS dataset

NGS data of 125 individuals publicly available from the JGA were reanalyzed using the same analysis pipeline to create the JGA-NGS dataset. BWA-0.7.16a was used to map read sequences to the GRCh37 reference genome, and GATK3.8.0-ge9d806836 was used for variant calling^4^. For the disease groups, only sequence data derived from DNA extracted from nontumor tissues or peripheral blood cells were subjected to reanalysis.

#### Variant normalization

The following normalization was performed for all variants in TogoVar. (1) The genomic coordinate system of the variants was standardized using GRCh37. The positions of the JGA-SNP variants were based on the dbSNP rs number for each probe of the SNP arrays. For GEM-J WGA, HGVD, JGA-NGS, and ToMMo 8.3KJPN, the GRCh37 positions described in the original data were used. (2) The notation of chromosome numbers (e.g., ChrM, ChrMT, MT, and 26) was standardized. (3) When the reference allele did not match the base of the positive strand of GRCh37, both reference and alternative alleles were converted to complementary bases. (4) The notation of alleles for insertion and deletion, which differ among analysis methods, was unified in the VCF format. (5) Normalization (parsimony and left alignment) of insertion and deletion was performed to eliminate the inconsistency between notations caused by an alignment method to the reference genome with the bcftools norm command^20^. (6) Variants with the same start position, reference allele, and alternative allele on GRCh37 were determined to be identical variants.

To reduce the risk of individual identification, we filtered out variants with allele counts of less than six in JGA-NGS and JGA-SNP. The union set of autosomal variant datasets from five Japanese populations (GEM-J WGA, HGVD, JGA-NGS, JGA-SNP, and ToMMo 8.3KJPN) was referred to as JPN_UNION (Table 1).

**Table 1:** JPN_UNION: The combined autosomal variant dataset of Japanese populations collected in TogoVar.

Dataset	Sample size	Platform	The number of autosomal variants
GEM-J WGA	7609	WGS	92,311,243
JGA-NGS	125	WES	4,561,009
JGA-SNP	183,884	SNP array	1,236,461
ToMMo 8.3KJPN	8380	WGS	91,462,814
HGVD v2.3	1208	WES	539,980
JPN_UNION	201,206		116,006,352

JPN_UNION included no duplicates in the number of autosomal variants, while it included duplicates in the sample size. More than half of the GEM-J WGA samples overlapped with ToMMo 8.3KJPN. Quality filters for variant calling defined for each dataset were not considered (i.e., all detected variants were counted). Additionally, multiallelic variants were decomposed into two or more monoallelic variants and considered as separate variants.

#### GRCh38 position of the variants

JGA-NGS, JGA-SNP, GEM-J WGA, and HGVD were transferred from GRCh37 to the GRCh38 reference sequence with CrossMap^21^. Some of the variants were not transferred to GRCh38. The gnomAD v2.1.1 and ToMMo 14KJPN datasets, which were obtained by variant calling on the GRCh38 reference sequence, were downloaded from their original sites. Note that the GRCh37-based data were used for tables, figures, and examples in this paper.

#### TogoVar ID assignment to JPN_UNION

The variants observed in JPN_UNION were assigned TogoVar IDs (e.g., tgv47264307), which permanently guaranteed the identity of the variants even when the reference sequence of the human genome was updated. The dbSNP rs number is a persistent ID; however, 50,497,907 variants in JPN_UNION have not yet been registered in dbSNP. Therefore, we assigned an original ID to each variant of JPN_UNION. In contrast to the dbSNP rs number, a TogoVar ID is assigned to each alternative allele; therefore, when two or more alternative alleles exist, it is possible to determine which specific allele is associated with phenotypes.

#### Variant annotation with Variant Effect Predictor

We used Variant Effect Predictor (VEP)^22^ to annotate variant types (SNV, insertion, deletion, indel, and substitution), variant consequences, SIFT^23^ and PolyPhen-2^24^ scores, gene symbols, and dbSNP rs numbers for the variants in JPN_UNION, gnomAD^7^, and ClinVar^9^.

#### Data annotation in the form of a knowledge graph

For the interpretation of variants, it is helpful to integrate annotations from well-curated biomedical databases. We have previously developed an integrated knowledge base from biomedical databases such as ClinVar in the Med2RDF project^25^, producing regularly updated knowledge graphs with the Resource Description Framework (RDF)^26^. Therefore, we converted the allele frequency data from VCF into RDF and developed SPARQL queries to obtain integrated results for the TogoVar database. The fact that such annotations are often embedded in the INFO field of the VCF files is neither scalable nor sufficient, as the required information varies depending on the needs of researchers. In addition, because there are no standards for encoding annotations in the INFO field, it could be complicated to utilize embedded information and update annotations when the new version of the external database is released. Instead, by aggregating variant information from VCF files and knowledge from various biomedical databases in RDF, we can easily extend the coverage of annotations and keep them current, which is a reasonable approach from the viewpoint of database management (Table 2).

**Table 2:** Knowledge graphs (RDF datasets) integrated in TogoVar.

Dataset	Description	RDF generation method	Method
ClinVar	Variants with clinical significance	Original XML files were converted with an RDF converter available at https://github.com/med2rdf/clinvar. Note that only variants whose position in GRCh37 was determined were loaded in TogoVar.	(1)
Colil^41^	Citation relationships in life sciences literature	TogoVar queries the Colil SPARQL endpoint at http://colil.dbcls.jp/sparql.	(2)
Ensembl	Transcripts of human genes	Data obtained from bioMart were converted with an RDF converter developed by us.	(4)
gnomAD	Allele frequencies in a variety of large-scale populations	Original VCF files were converted with vcf2rdf available at https://github.com/togovar/vcf2rdf.	(4)
GWAS Catalog	Genome-wide association studies	Original files were converted with an RDF converter developed by us.	(4)
HGNC	Approved gene names and symbols	Original files were converted with a converter available at https://github.com/med2rdf/hgnc.	(3)
Human Chromosome Ontology	Stable URIs for human reference genome versions	Original files were converted with a converter available at https://github.com/med2rdf/hco.	(3)
JPN_UNION	Allele frequencies in the Japanese population	Original VCF or tsv files were converted with vcf2rdf or RDF converters developed by us.	(4)
PubMed	Citations for biomedical literature	Original XML files were converted with an RDF converter developed by us.	(4)
PubTatorCentral^42^	Information on biomedical literature in which the names of variants appear	Original files were converted with an RDF converter developed by us. Used together with the bibliographic information of LitVar^43^ provided by Web API.	(4)
Sequence ontology^44^	Set of terms and relationships used to describe the features and attributes of biological sequence	Download an OWL file from http://www.ebi.ac.uk/ols/api/ontologies/so.	(1)

Four methods to retrieve RDF data: (1) importing public RDF files, (2) querying public SPARQL endpoints, (3) using converters published by Med2RDF, and (4) creating our proprietary converter to convert original files to RDF.

#### System architecture

**Fig. 1:**
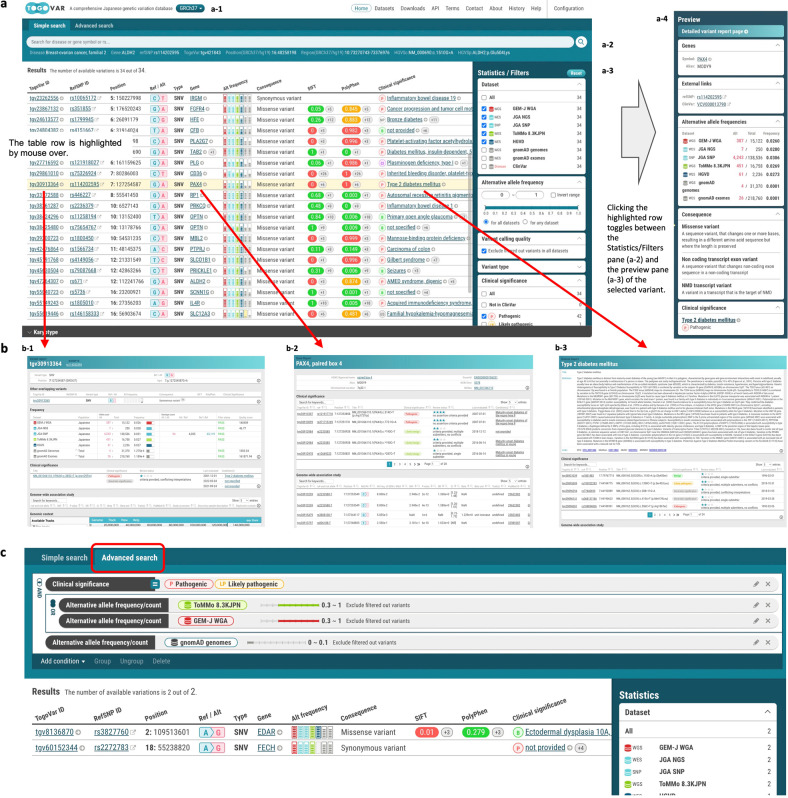
Overview of the TogoVar web interface. **a** Simple search. **a-1** Switch the reference sequence. **a-2** Text box for keyword search. Users can enter the following keywords: (1) position or range in the GRCh37 reference genome of a target variant, (2) dbSNP rs number, (3) TogoVar ID, (4) gene symbol including aliases, or (5) disease name (ClinVar condition). **a-3** Statistics/filters pane. Users can narrow down the search result by (1) dataset name, (2) alternative allele frequency, (3) type of variant (SNV, indel, etc.), (4) variant calling quality, and (5) clinical significance. The number of variants per facet is displayed. **a-4** Preview pane for the variant selected in the result table. **b** Report pages. **b-1** Variant report page. **b-2** Gene report page. **b-3** Disease report page. **c** Advanced search, showing search criteria for variants that have a GEM-J WGA or ToMMo 8.3KJPN allele frequency of 0.3 or higher and a gnomAD non-Finnish European allele frequency of 0.01 or lower and are pathogenic or likely pathogenic.

Report pages of TogoVar (Fig. 1b) consist of several modular components developed with the TogoStanza framework^27^ (http://togostanza.org/), which visualizes various information, such as allele frequencies, clinical significance, and literature. Information to be visualized in TogoStanza is retrieved from an RDF database with a query written in the SPARQL language. SPARQList (https://github.com/dbcls/sparqlist) was used to execute SPARQL queries and postprocess the results for a web application. These open-source applications provide high extensibility, allowing developers to create information retrieval queries and visualization components independently and embed them to a web page in combination. We used Virtuoso (https://virtuoso.openlinksw.com/) as the database management system for RDF data, Elasticsearch (https://www.elastic.co/) for keyword and faceted searches, and Ruby on Rails (https://rubyonrails.org/) as the web application framework.

## Results

### TogoVar web interface

**Fig. 2:**
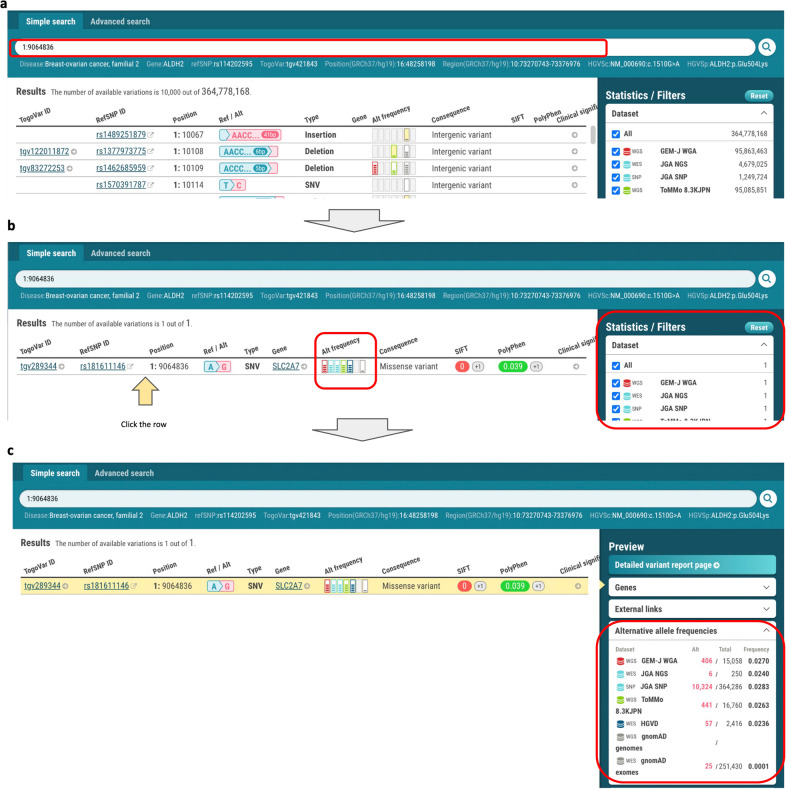
Search for variants by genomic position. **a** Genomic position (1:9064836) is entered as the search criteria. **b** A variant, tgv289344, satisfying the criteria is displayed, and the allele frequencies of tgv289344 in the datasets shown in the Filters pane are illustrated in the bar graphs. **c** The allele frequency of the selected tgv289344 is displayed numerically in the preview pane.

**Fig. 3:**
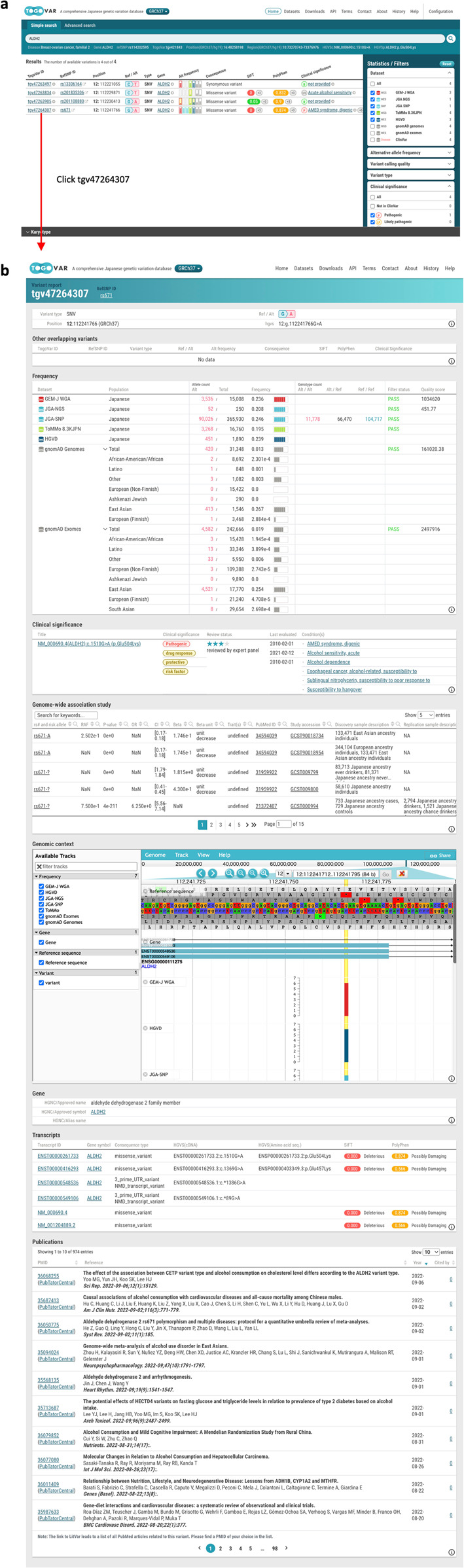
Examples of TogoVar search results by keywords and facets. **a** Search keyword input, facet filtering, and display of the number of entries per facet. **b** Variant report page.

In the TogoVar web interface, users can browse a list of variants in a tabular form retrieved by the keyword search and the faceted filtering interface (Fig. 1a) and switch between the GRCh38-based and GRCh37-based sites by selecting the button displaying the reference genome version in the header (Fig. 1a-1). Each column in the resulting table shows a summary of each variant, including a genomic position, a reference and an alternative allele, alternative allele frequencies derived from each dataset, annotations such as molecular consequences, deleterious effects predicted by SIFT and PolyPhen for a coding variant, and a clinical significance derived from ClinVar where available. Alternative allele frequencies are illustrated with a small bar chart, enabling users to grasp the difference in frequencies between the datasets. By clicking a TogoVar ID in the table, the variant report page describing detailed information is shown (Fig. 1b-1). Similarly, gene symbols and disease names are linked to a gene and disease report page, respectively (Fig. 1b-2, b-3). In addition, the advanced search interface allows users to make queries with more complex search criteria (Fig. 1c). Specifically, in addition to the variant types, consequences, gene symbols, and clinical significances, users can specify a different threshold for the allele frequency of each population using any combination of AND or OR operators to build nested complex queries interactively. Examples of these searches are presented in the following sections.

### Search by genomic position

Users can search for variants of interest based on the genomic position of the variant and compare the frequencies among datasets. Suppose that a researcher studying a rare disease and looking for disease-associated variants wants to know the frequency of a candidate variant in Japanese and other populations. The variant is located at the 9,064,836th base on chromosome 1, the reference allele is A, and the alternative allele is G. The researcher searches for the variant by position and finds tgv289344 (Fig. 2a). The alternative allele frequency in each dataset is shown in the bar graph icon, where the frequency in the rightmost dataset, gnomAD, was low (Fig. 2b). The preview panel shows the difference between the Japanese populations and the gnomAD populations numerically. The frequencies in the five Japanese populations are between 0.02 and 0.03, indicating that there is little difference in frequency among the Japanese populations (Fig. 2c).

### Search by variant annotations

Each variant in TogoVar is annotated with a molecular consequence, pathogenicity, deleterious prediction, and literature information in addition to alternative allele frequency. Users can search for variants based on these annotations. Suppose a researcher uses the following three criteria for searching variants: (1) variants in the ALDH2 gene, (2) variants with reference/alternative alleles in at least one Japanese population, and (3) variants with ClinVar clinical significance. At present, four variants have been identified (Fig. 3a). Among them, tgv47264307 (rs671), which has a high frequency of an alternative allele in the East Asian population and is associated with alcohol metabolism, is included.

The frequency table in the report page for tgv47264307 (Fig. 3b) shows the number of alleles and genotypes that are not displayed in the preview pane of the Simple search page and the QC results of variant calling for NGS-derived variants. The alternative allele frequency of tgv47264307 is approximately 0.2 in the Japanese and East Asian populations in gnomAD, while it is approximately 1 × 10^−5^ in the European and African populations, indicating a higher allele frequency in East Asia, including the Japanese population. The table of clinical significance shows an association with acute alcohol sensitivity in ClinVar. The table of the genome-wide association study includes a significant association between alcohol metabolism and alternative alleles in the East Asian population, including the Japanese population^28^. The transcripts table includes the results of molecular consequence and deleterious prediction for each transcript. The variant tgv47264307 is a missense variant predicted to affect the function of a protein according to the results of SIFT and PolyPhen-2. The related literature information is displayed at the bottom of the page, starting with the most recent, along with the number of citations.

### Bulk download and API access to TogoVar

The allele frequencies for each population and their annotations are downloadable in VCF format for GEM-J WGA or tab-delimited files for the other variant datasets from the download page^29^. Details are described in the README file that accompanies the download files in each directory. Terms of use are different for each variant dataset. Users should refer to the terms page of TogoVar^30^. The RESTful search API provides displayed contents in the simple and advanced search in JSON format^31^. The API specification is described in the Swagger framework.

### Comparison of JPN_UNION with gnomAD

**Fig. 4:**
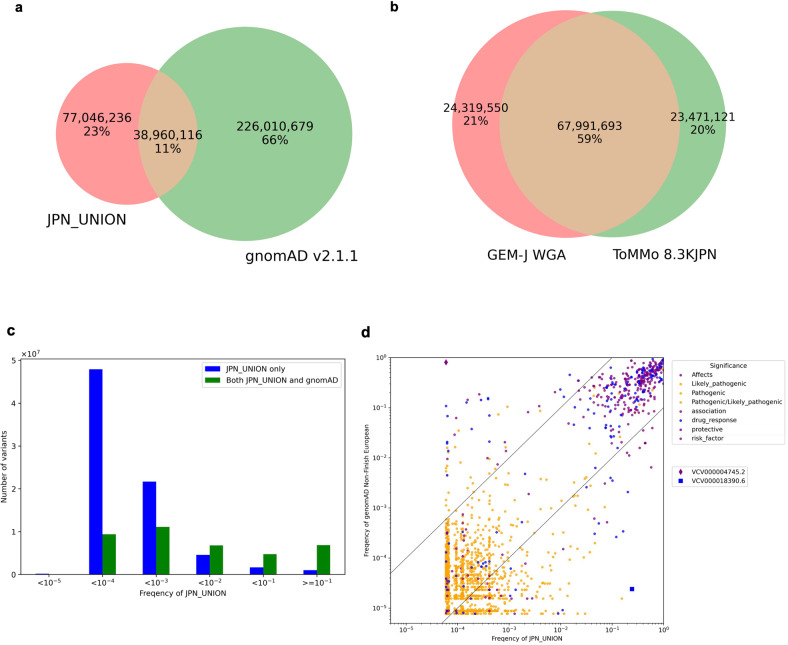
Comparison among variant datasets. **a** Comparison between JPN_UNION and gnomAD v2.1.1. gnomAD is a union set of the gnomAD exome and genome. **b** Comparison between GEM-J WGA and ToMMo 8.3KJPN. **c** Alternative allele frequency distribution of JPN_UNION. **d** Alternative allele frequency distribution of JPN_UNION and gnomAD non-Finnish European (NFE) variants limited to those with the following clinical significance in ClinVar: pathogenic, likely pathogenic, pathogenic/likely pathogenic, drug response, risk factor, association, affects, and protective. Variants outside the black line have a frequency ratio of 10 times or more. The number of alleles of NFE is the sum of those from the gnomAD exome and genome. The minimum frequency of JPN_UNION was 5.97 × 10^−5^ for a singleton variant in ToMMo 8.3KJPN (*n* = 8380, allele number = 16,760), the sample size of which was the second largest. JGA-SNP (*n* = 183,884, allele number = 367,768), with the largest sample size, targeted common variants and did not contain variants with a frequency of 0.01 or less.

To confirm the difference between variant datasets aggregated from the Japanese and non-Japanese populations, we counted the number of autosomal variants shared between JPN_UNION and gnomAD v2.1.1. The total number of autosomal variants in JPN_UNION and gnomAD v2.1.1 was 342,017,031, 11% of which were shared; 23% were included only in JPN_UNION. More than 77 million variants in TogoVar are not included in gnomAD (Fig. 4a). In addition, a comparison of GEM-J WGA and ToMMo 8.3KJPN, generated from WGS and accounting for the majority of JPN_UNION variants, showed that 59% of the variants were shared, 21% were included in GEM-J WGA only, and 20% were included in ToMMo 8.3KJPN only, indicating that there is a difference within the Japanese populations even though more than half of the samples overlap between GEM-J WGA and ToMMo 8.3KJPN (Fig. 4b). We compared the alternative allele frequency distribution of the variants included in JPN_UNION only with the distribution of those in both JPN_UNION and gnomAD (Fig. 4c). Figure 4c shows that the variants included in JPN_UNION only have a lower frequency than those included in both sets. Integration of variant datasets from multiple data sources in TogoVar enables an overview of variant data from various viewpoints.

### Integration of variant frequencies with clinical significance

Variants with different allele frequencies in different populations have been reported to have different pathogenicities^32^. To obtain an overall picture, we compared the allele frequencies of variants that are classified as pathogenic, likely pathogenic, pathogenic/likely pathogenic, drug response, risk factor, association, affects, and protective in ClinVar between JPN_UNION and gnomAD non-Finnish European (NFE). There were two clusters of high allele frequency and low allele frequency variants, and there were frequency differences between the two populations in low allele frequency variants. Variants with more than 10,000-fold frequency differences included those associated with skin/hair/eye pigmentation (VCV000004745.2), alcohol metabolism, and esophageal cancer (VCV000018390.6) (Fig. 4d).

## Discussion

In TogoVar, we integrated the allele frequency data of the five Japanese populations named JPN_UNION in Table 1 and found more than 77 million autosomal variants not included in gnomAD (Fig. 4a). We compared the alternative allele frequency distribution between JPN_UNION and gnomAD and found that variants present only in JPN_UNION were more likely to have low allele frequencies (Fig. 4c). This result indicates that gnomAD is insufficient for analyzing Japanese populations and that population-specific frequency databases such as TogoVar are important, especially when low allele frequency variants need to be considered.

In addition, we found that GEM-J WGA had 24,319,550 unique variants not included in ToMMo 8.3KJPN (Fig. 4b). This result shows that there is still room for discovering more novel variants in the Japanese population, and it is worthwhile to increase the Japanese sample size. However, GEM-J WGA and ToMMo 8.3KJPN have already aggregated several thousand Japanese individuals. We expect that novel variants will be found in the JGA-NGS dataset, as NGS data are continuously accumulating in the JGA.

In addition, by integrating multiple allele frequency datasets and their annotations, we confirmed the difference in allele frequencies between Japanese and European populations for variants interpreted as pathogenic in ClinVar (Fig. 4d). TogoVar searches for variants that match the search criteria and provides an overall view of the variant dataset through the annotation information. For example, users can confirm how many variants have clinical significance per dataset in the statistics/filter pane. Only 123,160 variants were found in JPN_UNION among 1,052,579 variants in ClinVar (Supplementary Fig. 1a, b). This result means that there are few cases where the clinical significance of ClinVar can be directly applied to variants in the Japanese population. The Medical Genomics Japan Variant Database (MGeND)^33^, which collects variant information with clinical significance from genome cohort projects for the Japanese population, is expected to fill this gap.

For 3 years, since the release of TogoVar, it has been used as reference data for the allele frequency of germline variants in the Japanese population. TogoVar is becoming recognized as shared infrastructure data. The purposes for its use are variant prioritization^34^, determination of whether a variant of interest is known or unknown^35^, and comparison of allele frequencies in TogoVar with those of disease groups or non-Japanese populations^36,37^.

One shortcoming of TogoVar is the lack of features to confirm how individual-level read sequences where variants are detected are mapped to the reference genome. In particular, the mapped reads around the variants in genetic research for rare diseases are crucial for assessing whether the variants are accurately detected. gnomAD visualizes the mapping of the reads. In contrast, individual-level read sequence data are an individual identification code defined in the Personal Information Protection Law of Japan^38^, which prohibits the publication of the reads in a way that reveals the individual sequence. Instead, we plan to display an average depth per base and statistical information without this restriction. We also provided links to individual-level NGS datasets from which the variants were detected (Supplementary Fig. 2). Although users are required to apply to the NBDC DAC to download individual-level NGS data from the JGA^3^, they can confirm the read mapping quality and perform joint calling with their own NGS data.

The variant data collection section described that GEM-J WGA, JGA-NGS, and JGA-SNP were generated from multiple JGA datasets. To check the read mapping around the variants of interest in GEM-J WGA, users need to download all six JGA datasets generated from the GEM-J WGA. Thus, it is not possible to know which JGA dataset(s) contain the variants of interest from the information currently available at TogoVar. The frequency of each JGA dataset can be used as unrestricted data to solve this problem. However, attribution disclosure attacks via the DNA (ADAD) method^39^ make it possible to estimate whether a target individual is included in the frequency dataset if an attacker has a genome sequence of the individual. Thus, it can be inferred that the target individual is affected by the disease from an allele frequency dataset consisting of only individuals affected by a single disease. To avoid this problem, we considered implementing registered access^40^, which is an access control method of intermediate strength between controlled-access and unrestricted access. By implementing this registered access, researchers can identify which JGA dataset to request and will not need to apply for and download unnecessary JGA datasets.

There are many candidate datasets to be added to TogoVar, such as linkage disequilibrium data, structural variants, multiomics data, and clinical significance data in the Japanese population from MGeND^33^, to prioritize and interpret variants. We will integrate these data to contribute to the elucidation of the association between variants and traits.

## Supplementary information

Supplementary information

## References

[1] Tryka KA (2014). NCBI’s database of genotypes and phenotypes: dbGaP. Nucleic Acids Res..

[2] Lappalainen I (2015). The European Genome-phenome Archive of human data consented for biomedical research. Nat. Genet..

[3] Fukuda A, Kodama Y, Mashima J, Fujisawa T, Ogasawara O (2021). DDBJ update: streamlining submission and access of human data. Nucleic Acids Res..

[4] DBCLS. How the JGA dataset was generated. https://togovar.org/doc/datasets/analysis. Accessed 2 November (2022).

[5] Tadaka S (2020). jMorp updates in 2020: large enhancement of multi-omics data resources on the general Japanese population. Nucleic Acids Res..

[6] Higasa K (2016). Human genetic variation database, a reference database of genetic variations in the Japanese population. J. Hum. Genet..

[7] Karczewski KJ (2020). The mutational constraint spectrum quantified from variation in 141,456 humans. Nature.

[8] Phan, L. et al. ALFA: Allele frequency aggregator. https://www.ncbi.nlm.nih.gov/snp/docs/gsr/alfa/. Accessed 2 November (2022).

[9] Landrum MJ (2018). ClinVar: improving access to variant interpretations and supporting evidence. Nucleic Acids Res..

[10] Buniello A (2019). The NHGRI-EBI GWAS Catalog of published genome-wide association studies, targeted arrays and summary statistics 2019. Nucleic Acids Res..

[11] DBCLS. TogoVar Datasets. https://togovar.org/doc/datasets/. Accessed 2 November (2022).

[12] DBCLS. JGA-NGS dataset in TogoVar. https://togovar.org/doc/datasets/jga_ngs. Accessed 2 November (2022).

[13] DBCLS. JGA-SNP dataset in TogoVar. https://togovar.org/doc/datasets/jga_snp. Accessed 2 November (2022).

[14] Nagai A (2017). Overview of the BioBank Japan Project: Study design and profile. J. Epidemiol..

[15] Japan Agency for Medical Research and Development. GEM Japan (GEnome Medical alliance Japan). https://www.amed.go.jp/en/aboutus/collaboration/ga4gh_gem_japan.html. Accessed 2 November (2022).

[16] DBCLS. GEM-J WGA dataset in TogoVar. the GEnome Medical alliance Japan (GEM Japan) project https://togovar.org/doc/datasets/gem_j_wga#jga_datasets. Accessed 2 November (2022).

[17] Kuriyama S (2016). The Tohoku Medical Megabank Project: Design and mission. J. Epidemiol..

[18] NBDC. hum0103-v3 in the NBDC human database. https://humandbs.biosciencedbc.jp/en/hum0103-v3. Accessed 2 November (2022).

[19] Tadaka S (2019). 3.5KJPNv2: an allele frequency panel of 3552 Japanese individuals including the X chromosome. Hum. Genome Var..

[20] Danecek, P. et al. bcftools norm command. https://samtools.github.io/bcftools/bcftools.html#norm. Accessed 2 November (2022).

[21] Zhao H (2014). CrossMap: a versatile tool for coordinate conversion between genome assemblies. Bioinformatics.

[22] McLaren W (2016). The ensembl variant effect predictor. Genome Biol..

[23] Kumar P, Henikoff S, Ng PC (2009). Predicting the effects of coding non-synonymous variants on protein function using the SIFT algorithm. Nat. Protoc..

[24] Adzhubei IA (2010). A method and server for predicting damaging missense mutations. Nat. Methods.

[25] MED2RDF project. MED2RDF website. http://med2rdf.org/. Accessed 2 November (2022).

[26] RDF Working Group. RDF - Semantic Web Standards. https://www.w3.org/RDF/. Accessed 2 November (2022).

[27] Katayama T (2019). TogoGenome/TogoStanza: modularized Semantic Web genome database. Database.

[28] Matoba N (2020). GWAS of 165,084 Japanese individuals identified nine loci associated with dietary habits. Nat. Hum. Behav..

[29] DBCLS. TogoVar Downloads. https://togovar.org/downloads/. Accessed 2 November (2022).

[30] DBCLS. Terms in TogoVar. https://togovar.org/doc/terms. Accessed 2 November (2022).

[31] DBCLS. TogoVar API. https://togovar.org/api. Accessed 2 November (2022).

[32] Manrai AK (2016). Genetic misdiagnoses and the potential for health disparities. N. Engl. J. Med..

[33] Kamada M (2019). MGeND: an integrated database for Japanese clinical and genomic information. Hum. Genome Var..

[34] Kiso M (2020). Clinical and genomic characteristics of mucosal signet-ring cell carcinoma in Helicobacter pylori-uninfected stomach. BMC Gastroenterol..

[35] Kato K, Ozawa T, Ohno S, Nakagawa Y, Horie M (2020). Postoperative supraventricular tachycardia and polymorphic ventricular tachycardia due to a novel SCN5A variant: a case report of a rare comorbidity that is difficult to diagnose. BMC Cardiovasc. Disord..

[36] Lee I-H, Lee J-W, Kong SW (2020). A survey of genetic variants in SARS-CoV-2 interacting domains of ACE2, TMPRSS2 and TLR3/7/8 across populations. Infect. Genet Evol..

[37] Isshiki T (2021). Association of rs3750920 polymorphism in TOLLIP with clinical characteristics of fibrosing interstitial lung diseases in Japanese. Sci. Rep..

[38] Personal Information Protection Commission, Japan. Amendment to the Cabinet Order to Enforce the Act on the Protection of Personal Information (Tentative Translation). https://www.ppc.go.jp/files/pdf/Cabinet_Order.pdf. Accessed 2 November (2022).

[39] Erlich Y, Narayanan A (2014). Routes for breaching and protecting genetic privacy. Nat. Rev. Genet..

[40] Dyke SOM (2018). Registered access: authorizing data access. Eur. J. Hum. Genet..

[41] Fujiwara T, Yamamoto Y (2015). Colil: a database and search service for citation contexts in the life sciences domain. J. Biomed. Semant..

[42] Wei C-H, Allot A, Leaman R, Lu Z (2019). PubTator central: automated concept annotation for biomedical full text articles. Nucleic Acids Res..

[43] Allot A (2018). LitVar: a semantic search engine for linking genomic variant data in PubMed and PMC. Nucleic Acids Res..

[44] Eilbeck K (2005). The Sequence Ontology: a tool for the unification of genome annotations. Genome Biol..

