# Supplementary material for: TogoVar: A comprehensive Japanese genetic variation database
Source: Hum Genome Var. 2022 Dec 12;9:44. doi: 10.1038/s41439-022-00222-9 (PMC9744889; doi:10.1038/s41439-022-00222-9)

Supplementary Fig.1 Number of variants per clinical significance

a In the Clinical significance box, the number of variants per clinical significance is displayed for 1,052,579 ClinVar variants in TogoVar. Note that the number is counted for each significance when two or more clinical significances are assigned to one variant.
b The number per clinical significance for 123,160 variants included in the five datasets of JPN_UNION is displayed.

a

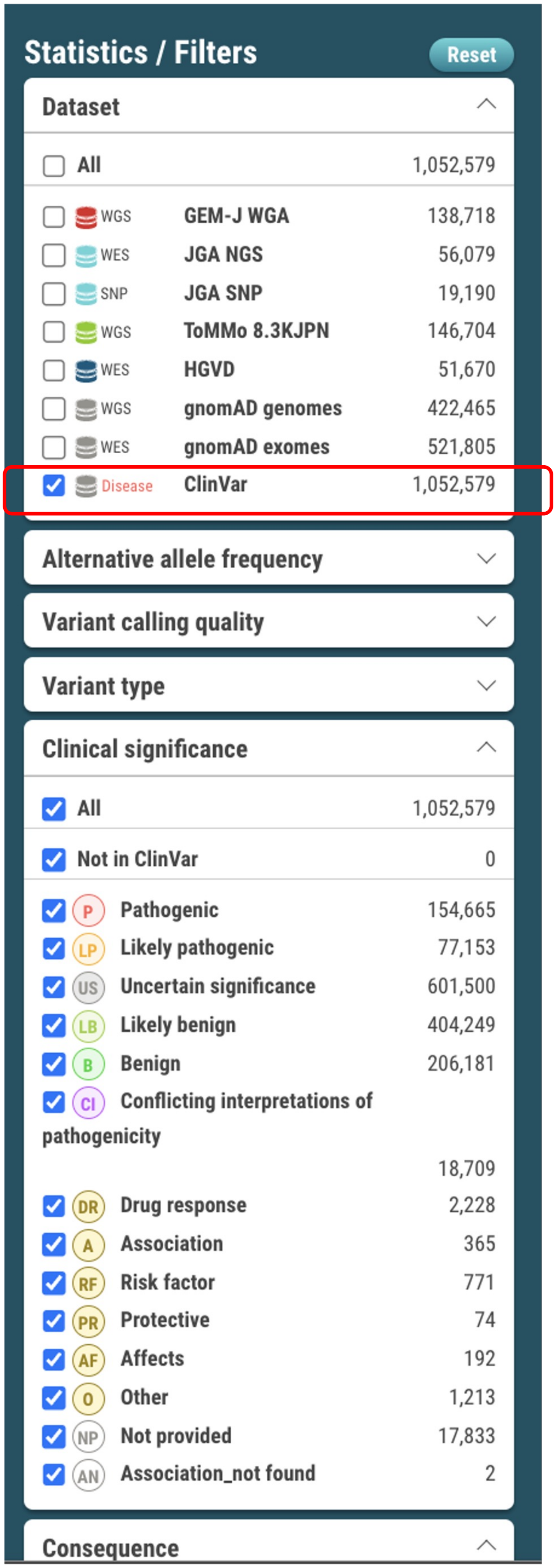

b

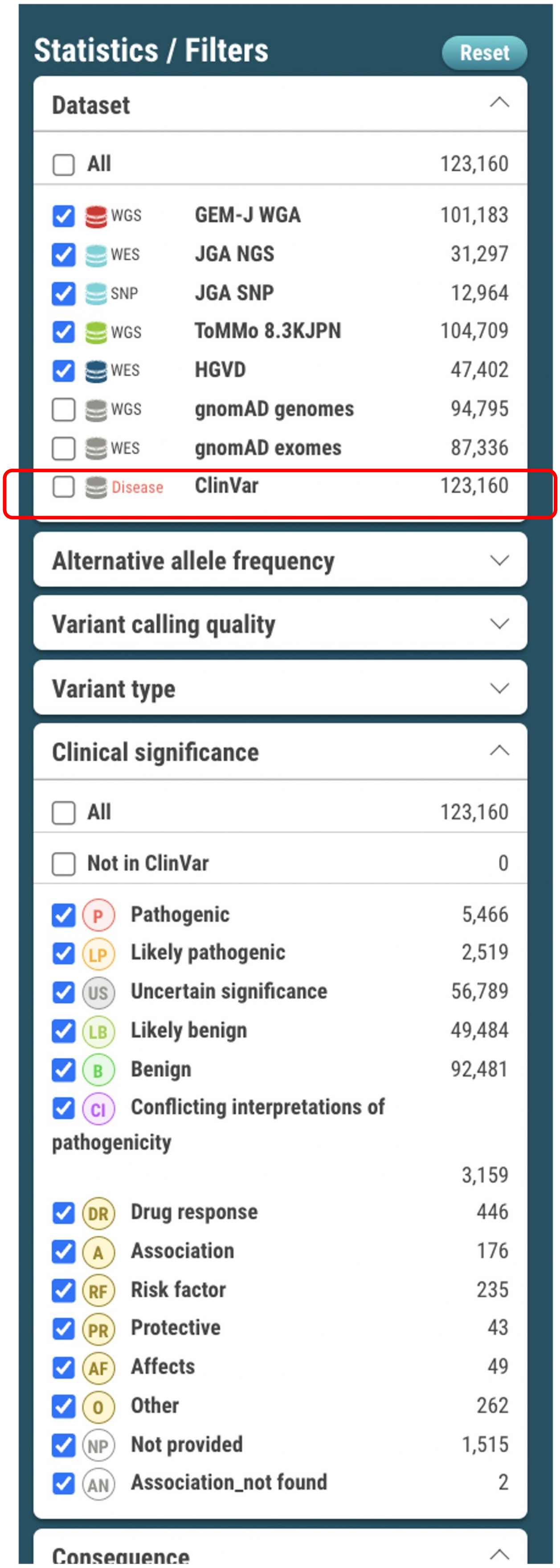

Supplementary Fig.2 How to access individual-level genome data in JGA

a Identify the allele frequency dataset (GEM-J WGA) that contains the variant of interest. **b** Focus on hum0184 among the six individual-level genomic datasets of GEM-J WGA. **c** Obtain an overview of the hum0184 dataset in the NBDC human database. d Submit a data-use application.

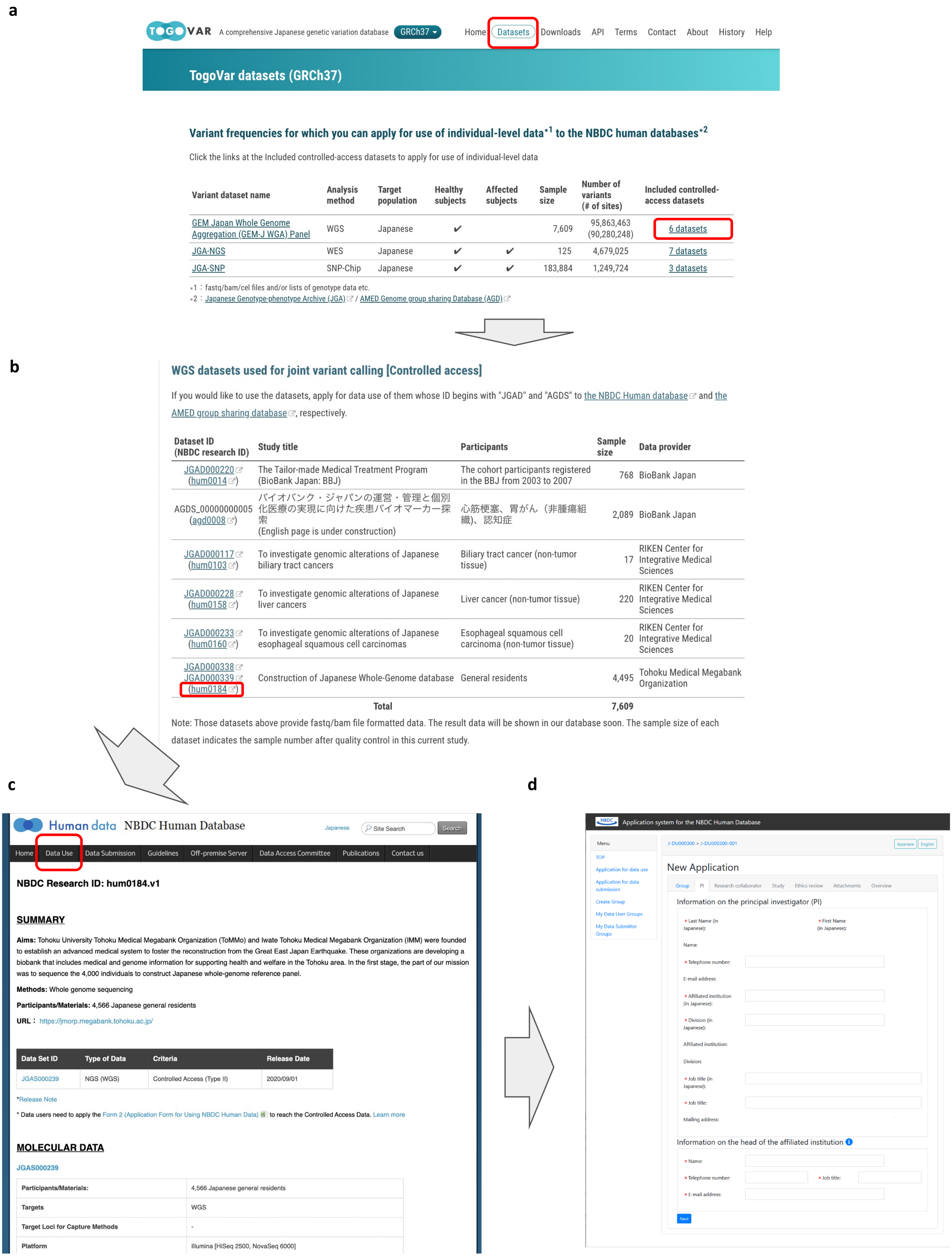

Supplement: Supplementary file 1 — Supplementary information [file 41439_2022_222_MOESM1_ESM.pdf]
